# Blocks identical by descent in the genomes of the indigenous population of Siberia demonstrate genetic links between populations

**DOI:** 10.18699/VJGB-23-08

**Published:** 2023-03

**Authors:** N.A. Kolesnikov, V.N. Kharkov, K.V. Vagaitseva, A.A. Zarubin, V.A. Stepanov

**Affiliations:** Research Institute of Medical Genetics, Tomsk National Research Medical Center of the Russian Academy of Sciences, Tomsk, Russia; Research Institute of Medical Genetics, Tomsk National Research Medical Center of the Russian Academy of Sciences, Tomsk, Russia; Research Institute of Medical Genetics, Tomsk National Research Medical Center of the Russian Academy of Sciences, Tomsk, Russia; Research Institute of Medical Genetics, Tomsk National Research Medical Center of the Russian Academy of Sciences, Tomsk, Russia; Research Institute of Medical Genetics, Tomsk National Research Medical Center of the Russian Academy of Sciences, Tomsk, Russia

**Keywords:** IBD, human populations, Siberian populations, IBD, популяции человека, сибирские популяции

## Abstract

The gene pool of the indigenous population of Siberia is a unique system for studying population and evolutionary genetic processes, analyzing genetic diversity, and reconstructing the genetic history of populations. High ethnic diversity is a feature of Siberia, as one of the regions of the peripheral settlement of modern human. The vast expanses of this region and the small number of aboriginal populations contributed to the formation of significant territorial and genetic subdivision. About 40 indigenous peoples are settled on the territory of the Siberian historical and ethnographic province. Within the framework of this work, a large-scale population study of the gene pool of the indigenous peoples of Siberia was carried out for the first time at the level of high-density biochips. This makes it possible to fill in a significant gap in the genogeographic picture of the Eurasian population. For this, DNA fragments were analyzed, which had been inherited without recombination by each pair of individuals from their recent common ancestor, that is, segments (blocks) identical by descent (IBD). The distribution of IBD blocks in the populations of Siberia is in good agreement with the geographical proximity of the populations and their linguistic affiliation. Among the Siberian populations, the Chukchi, Koryaks, and Nivkhs form a separate cluster from the main Siberian group, with the Chukchi and Koryaks being more closely related. Separate subclusters of Evenks and Yakuts, Kets and Chulyms are formed within the Siberian cluster. Analysis of SNPs that fell into more IBD segments of the analyzed populations made it possible to compile a list of 5358 genes. According to the calculation results, biological processes enriched with these genes are associated with the detection of a chemical stimulus involved in the sensory perception of smell. Enriched for the genes found, molecular pathways are associated with the metabolism of linoleic, arachidonic, tyrosic acids and by olfactory transduction. At the same time, an analysis of the literature data showed that some of the selected genes, which were found in a larger number of IBD blocks in several populations at once, can play a role in genetic adaptation to environmental factors.

## Abstract (ru)

Генофонд коренного населения Сибири представляет собой уникальную систему с точки зрения исследования популяционно- и эволюционно-генетических процессов, анализа генетического разнообразия и реконструкции генетической истории популяций. Высокое этническое разнообразие является особенностью Сибири как одного из регионов периферийного расселения современного человека. Огромные пространства этого региона и малочисленность аборигенного населения способствовали формированию значительной территориальной и генетической подразделенности. На территории сибирской историко-этнографической провинции расселены около 40 коренных народностей. Проведено масштабное популяционное исследование генофонда коренных народов Сибири на уровне высокоплотного ДНК-микрочипа Infinium Multi-Ethnic Global-8, позволяющее заполнить существенный пробел в геногеографической картине населения Евразии. Для этого были отобраны и проанализированы фрагменты ДНК, унаследованные без рекомбинации каждой парой индивидов от их недавнего общего предка, т. е. сегменты (блоки), идентичные по происхождению (IBD). Распределение блоков IBD в популяциях Сибири хорошо согласуется с географической близостью популяций и их языковой принадлежностью. Чукчи, коряки и нивхи среди сибирских популяций формируют отдельный от основной группы Сибири кластер, причем чукчи и коряки являются более близкородственными. Образуются отдельные субкластеры эвенков и якутов, кетов и чулымцев, тувинцев и алтайцев внутри сибирского кластера. Анализ SNP, которые попадали в большее количество IBD-сегментов анализируемых популяций, позволил составить список из 5358 генов. По результатам расчета, обогащенные этими генами биоло- гические процессы связаны с обнаружением химического раздражителя, участвующего в сенсорном восприятии запаха. Обогащенные найденными генами молекулярные пути связаны с метаболизмом линолевой, арахидоновой, тирозиновой кислот и путем обонятельной трансдукции. При этом анализ литературных данных показал, что неко- торые из отобранных генов, которые встречались в большем количестве блоков IBD сразу в нескольких популяциях, могут играть роль в адаптации человека к факторам окружающей среды

## Introduction

Genetic and demographic processes in populations, population fluctuations, cross-breeding events, migrations and natural selection affect the structure of genetic diversity in the genomes of individuals and populations as a whole. In particular, genetic and demographic processes lead to the formation of linkage blocks of common origin (identity by descent, IBD). A segment having identical nucleotide sequences is IBD in two or more individuals if they have inherited it from a common ancestor without recombination, that is, in these individuals the segment has a common origin. The expected length of an IBD block depends on the number of generations that have passed since the segment appeared in the last common ancestor (Browning S.R., Browning B.L., 2010; Palamara et al., 2012).

IBD segments can be used to reveal the demographic history of populations, including bottleneck effects and gene flows in populations (Gusev et al., 2012). Recent studies have shown differences in IBD distribution between African, Asian, and European populations, as well as IBD segments shared with ancient genomes such as those of Neanderthals and Denisovans (Hochreiter, 2013).

Close relatives have rather long DNA fragments identical with each other and, accordingly, in most chromosomes there are blocks of considerable length identical by descent (> 66.7 cM), as a result of which the expected length of the total IBD is about 1700 cM. Cousins and second cousins are expected to have multiple regions (more than 2.5 expected segments) due to the presence of recent ancestors determining their relationship. For first cousins, each IBD is expected to have a total length greater than 62 cM and for second cousins, 25 cM. Distant cousins that are fourth cousins or more distant are very likely to carry one or more regions from their nearest common ancestor. Such couples include the vast majority of people in a particular population and are usually referred to as “unrelated” because the proportion and number of IBD across the genome is expected to be relatively small between them (Gusev et al., 2012).

IBD segments can also help in the detection of natural selection signals in the human genome. Searching for regions with an excess of IBD segments allows the identification of genomic regions in the human genome that are under very recent and strong selection, since selection generally increases the number of IBD segments among individuals in a population (Albrechtsen et al., 2010; Han, Abney, 2011).

Regarding the populations of the indigenous ethnic groups of Siberia, it has been suggested that large-scale dispersal and mixing of populations probably may explain the unusually high proportion of IBD between populations (Pugach et al., 2016).

The purpose of this study was to analyze the structure of the gene pool of populations of the indigenous population of Siberia, based on the identification of linkage blocks identical by descent, and their intra- and interpopulation distribution.

## Materials and methods

Genome-wide genotyping data were generated using Infinium Multi-Ethnic Global-8 microarrays (Illumina) with over 1.7 million markers. Samples with more than 5 % missing positions, as well as SNPs with more than 10 % missing genotypes, were excluded from the analysis. The data were preliminarily filtered by the minimum rare allele frequency (MAF, minor allele frequency > 0.01). As a result, 886,889 autosomal SNPs were included in the final data set

Populations of the indigenous peoples of Siberia (N = 477) are represented by Altaians (B – the village of Beshpeltir, Chemalsky district, N = 24 and K – the village of Kulada, Ongudaysky district, N = 25), Buryats (A – the village of Aginskoye, Aginsky district, N = 23 and K – the village of Kurumkan, Kurumkansky district, N = 28), Kalmyks (N = 29), Kets (N = 15), Koryaks (N = 20), Chukchi (N = 25). The Koryak material was collected in the Koryak Autonomous District of the Kamchatka Region. A population sample of the Chukchi whose blood samples were collected in the villages of Lorino, Sireniki, Yanarykot and Novoe Chaplino of the Chukotka Autonomous Okrug belongs to the coastal group, Nivkhs (N = 13), Tatars (T – Tomsk, N = 20), Tuvans (N = 28), Udeges (N = 15), Khantami (K – the village of Kazym, Beloyarsk district, N = 30 and R – the village of Russkinskaya, Surgut district N = 26), Khakases (T – Sagais of the Tashtyp district N = 29 and S – Kachins of the Shirinsky district N = 26), Chulyms (N = 22), Evenks (Z – Transbaikalian (Chara village of the Kalarsky district, Moklakan village and Tupik village of the Tungiro-Olyokma region) N = 25 and Y – Yakut Evenks (N = 28) and Yakuts (N = 26).

The material was deposited in the bioresource collection “Biobank of the Population of Northern Eurasia”. The characteristics of the studied populations are presented in Table 1.

**Table 1.**
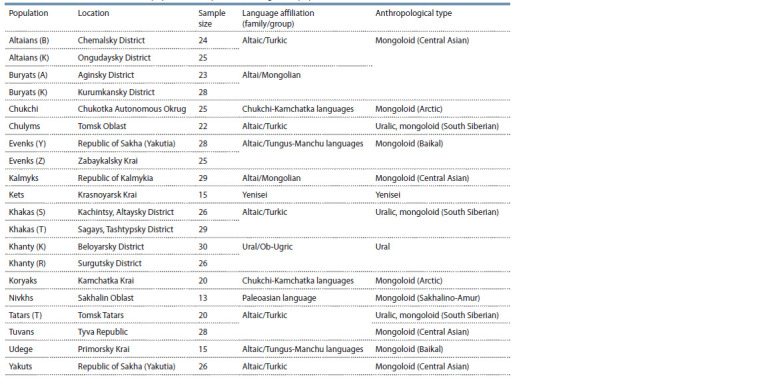
Characteristics of the studied population samples of the indigenous population of Siberia

Phasing of genotypes was carried out using Beagle 4.1 software (Browning S.R., Browning B.L., 2007). The Refined IBD algorithm, refined-ibd.16May19.ad5.jar version (Browning B.L., Browning S.R., 2013), was used to analyze genome blocks identical by descent. To compare the populations, the sums of the average lengths of the IBD segments between pairs of individuals were obtained for the following length ranges: 1.5–1.999 cM, 2–3.999, 4–7.999, 8–15.999 and >16 cM (for convenience, these ranges are referred to further in the text as 1.5–2 cM, 2–4, 4–8 and 8–16 cM). A heat map with a dendrogram based on the logarithm of the sum of the average IBD segment lengths between pairs of individuals was built using the heatmap.2 package in the R software environment

We also identified SNPs that fell into a larger number of IBD segments of the analyzed populations (the frequency of SNPs in IBD was higher than the 99th quantile of the frequency distribution), determined the belonging of these SNPs to genes, and assessed the biological significance of the resulting list of these genes. For this analysis, we used the WebGestalt web resource (WEB-based Gene SeT AnaLysis Toolkit); in particular, the analysis of KEGG paths and gene ontologies (Gene Ontology) was conducted using the ORA (over-representation analysis) method.

## Results and discussion

For a more detailed analysis of the genetic relationship of the Siberian populations and to find out to what extent their genetic structure can be explained by recent local migrations, we isolated and analyzed DNA fragments that were inherited without recombination by each pair of individuals from their recent common ancestor, that is, segments (blocks) identical by descent (IBD).

**Fig. 1.**
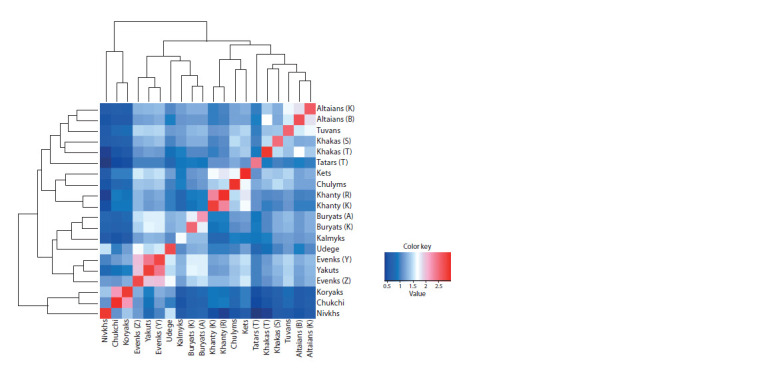
Heatmap with dendrogram based on the logarithm of the sum of mean IBD segment lengths (>1.5 cM) between pairs of individuals

The populations inhabiting the territory of Siberia are characterized by a unique genetic and demographic history, which is reflected, among other things, in the distribution of IBD blocks both within the populations and between them. We calculated the sums of the average lengths of the IBD segments between pairs of individuals and, based on their results, built a heat map with a dendrogram based on the logarithm of the sum of the average lengths of the IBD segments (Fig. 1).

The number of common segments among representatives of different populations is consistent with the geography of their residence, since the peoples living nearby can be influenced by common genetic and demographic processes. Analysis of the heat map demonstrates the clustering of the populations of the Siberian group, linking peoples by place of origin. Among the Siberian populations, the Chukchi, Koryaks and Nivkhs form a separate cluster from the main group of Siberian populations, with the Chukchi and Koryaks being more closely related. Separate subclusters of Evenks and Yakuts, Kets and Chulyms, Tuvans and Altaians are formed within the Siberian cluster.

With the gradation of the IBD segments with different mean length, the trend generally remains, but some differences appear that more accurately characterize the recent admixture between the peoples. For longer IBDs, the clusters are more in line with the current geographic location of the populations, reflecting the recent exchange of common regions. In three length ranges with IBD sizes (1.52–2, 2–4, and 4–8 cM), populations are better divided into closely geographically located pairs: Koryak-Chukchi, Yakut-Evenki The Kets almost equally share IBD blocks with the Chulyms (18.7–27.2–7.7 cM) and Khanty (23.4–24.4–4.8 cM for Khanty (K) and 25.9–30.1–7.9 cM for the Khanty (R)), while among the Khanty, a greater value of common IBD blocks is observed in the Russian Khanty, which corresponds to their closer geographical location compared to the Khanty of the Beloyarsky district.

For the Khakas, who are more distant from the Kets, the values of the total IBD blocks are almost two to three times lower than for the Khanty (9.8–10.4–2.5 cM for the Khakas (S) and 9.1–10.6–3.8 cM for Khakas (T)). Despite the fact that the Evenks, Tuvans and Yakuts are even more remote, for the Tuvans (12.3–12.8–2.1 cM) and the Yakuts (12.8–12.5–2.1 cM), there are similar values of total IBD blocks, for the Trans- Baikal Evenks (16.4–16.9–3.0 cM), it is worth noting that the values of the IBD blocks are greater than with the Yakut Evenks (14.7–14.0–2.6 cM).

With few exceptions, common IBD segments between Siberian populations are better explained by the geographic proximity of the populations rather than by their linguistic affiliation. For example, the Yakut Evenks living in the territory of Yakutia have more IBDs in common with the Yakuts (252.7 cM) than with the Transbaikal Evenks (102.5 cM). At the same time, the sum of the average lengths of IBD segments between pairs of individuals between two populations of the Evenks is comparable to the sum between the Transbaikalian Evenks and Yakuts (95.2 cM).

In terms of intrapopulation IBD analysis, in general, individuals from populations of the Far North and Far East (Koryaks, Chukchi, Nivkhs) share more IBDs with specimens from the same group than individuals from the South Siberian populations such as the Altaians and Tuvans. At the same time, in the Chukchi, Koryaks, and Nivkhs, short IBD fragments of 1.5–4 cM (55–57–59 %) make the largest contribution, which may indicate a bottleneck in the past during migrations to the north and northeast and/or a strong isolation from other populations inhabiting the territory of Siberia. At the same time, in the Chulyms, Khanty (R), inhabiting the central part of Siberia and having the largest sum of average lengths of IBD segments between pairs of individuals, the largest contribution is made by IBDs longer than 8 cM (47–51 %), which indicates a strong recent inbreeding within the population

**Fig. 2.**
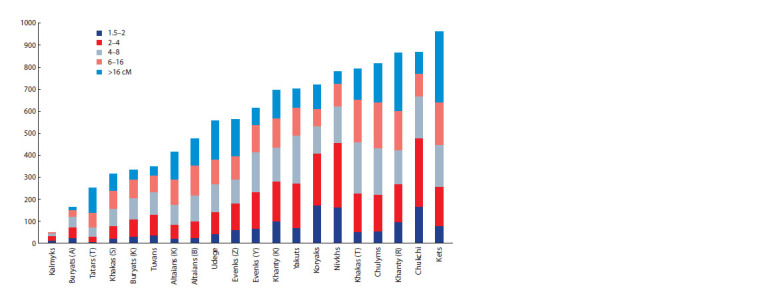
Diagram of the sum of the average lengths of IBD segments between pairs of individuals in the studied populations for IBDs of different sizes (1.5–2, 2–4, 4–8, 8–16, >16 cM).

The most genetically heterogeneous Siberian populations, which have minimal values for the genomic inbreeding coefficient (FROH) (Kolesnikov et al., 2021), also have minimal values for the sum of the average lengths of IBD segments. This is most pronounced in the populations of Kalmyks, Agin Buryats, and Tomsk Tatars (Fig. 2).

In Buryat populations, there are no significant differences in the average total length between pairs of individuals with other populations, but there is a significant difference in the distribution of IBD within populations. Thus, the Buryats (K) have a significantly larger average total length between pairs of individuals within the population (335.4 cM), compared with the Buryats (A) (163.5 cM), largely due to medium and long IBD. The Agin Buryats have a much higher proportion of short IBDs (16–30–28–19–7 %) than the Kurumkan Buryats (9–24–29–25–13 %). Despite the fact that for the Buryats, who have a large total population, with their numbers almost doubling from 237 thousand in 1926 to 461 thousand in 2010, this difference between populations can be explained by a sharp increase in the population of the village of Aginskoye from 451 people in 1908 up to 4556 people in 1939 and 15 thousand in 2010, and the stability of the population of the village of Kurumkan, with a population of 5617 people in 1979 and 5465 in 2010, located in one of the remote regions of Buryatia.

A similar dynamics is observed in the populations of the Chukchi (20–36–22–12–11 %), Koryaks (24–33–17–11– 15 %), Nivkhs (21–38–21–13–7 %) and Kalmyks (29–39– 22–7–3 %), followed by a sharper decrease in the proportion of long IBD. The Chukchi, Nivkhs and Koryaks, which have a small population (up to 13 thousand), are characterized by the absence of sharp fluctuations in the number of populations over the past hundred years with an increase of 29–9–6 %, respectively, which could also contribute to a reduction in the total long IBD segments subject to a small number of closely related marriages within the population

A total of 189,314 SNPs were obtained for 20 populations, falling into the largest number of IBD segments of the analyzed populations (the frequency of SNPs falling into IBD is higher than the 99th quantile of the frequency distribution). Of these, 88,530 SNPs are located in intergenic regions, the rest are located in the region of 5358 genes. Table 2 shows the genes that were shown in four or more populations.

**Table 2.**
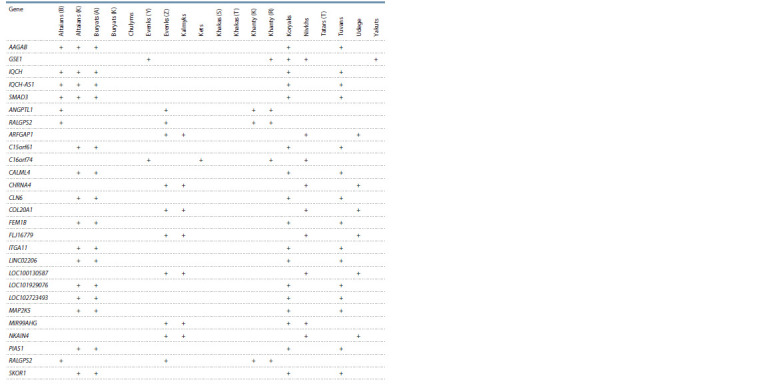
Genes falling into regions with the maximum number of IBD blocks in more than four populations

From the list of 5358 genes most frequently found in IBD blocks, 1694 were annotated using the KEGG database according to WebGestalt. As a result, analysis taking into account the Benjamini–Hochberg correction (FDR = 0.05) revealed molecular KEGG pathways enriched in these genes: the linoleic acid pathway hsa00591 (FDR = 0.0051) including 17 genes (CYP2C8, CYP2C9, PLA2G1B, PLB1, CYP1A2, CYP2C19, CYP3A4, PLA2G10, PLA2G2A, PLA2G2C, PLA2G2D, PLA2G2E, PLA2G2F, PLA2G4A, PLA2G4C, PLA2G5, PLA2G6), the arachidonic acid pathway hsa00590 (FDR = 0.0240) including 27 genes (CYP2C8, CYP2C9, PLA2G1B, PLB1, ALOX12, ALOX12B, ALOX15B, ALOX5, CYP2B6, CYP2C19, GPX1, GPX3, GPX7, PLA2G10, PLA2G2A, PLA2G2C, PLA2G2D, PLA2G2E, PLA2G2F, PLA2G4A, PLA2G4C, PLA2G5, PLA2G6, PTGIS, PTGS1, PTGS2, TBXAS1), tyrosine metabolism pathway hsa00350 (FDR = 0.0240) including 18 genes (ADH1A, ADH1B, ADH1C, ADH4, ADH5, ADH6, ADH7, ALDH3B1, ALDH3B2, AOC2, AOC3, DDC, GOT1, HPD, IL4I1, PNMT, TYR, TYRP1), olfactory transduction pathway hsa04740 (FDR = 4.55E-08) including 159 genes.

The metabolic conversion of polyunsaturated fatty acids (PUFAs) such as linoleic acid into biologically active long chain PUFAs (> 20 carbons, LC-PUFAs) such as arachidonic acid is essential for proper metabolism. LC-PUFAs and their metabolites are important structural and signaling components for numerous biological systems, including brain development and function, innate immunity, and energy homeostasis (Marszalek, Lodish, 2005; Calder, 2013). There are also food sources of preformed LC-PUFAs in eggs and some meats containing arachidonic acid (Horrocks, Yeo, 1999; Howe et al., 2006; Chilton et al., 2014). The patterns found in the distribution of IBD containing genes involved in fatty acid metabolism may indicate recent directional selection associated with adaptation to dietary habits in cold climates or reflect the influence of a Western diet (Chilton et al., 2014).

Positive selection in genes that affect the level of LC-PUFAs, as well as the metabolic efficiency by which LC-PUFAs are formed in populations of the Pygmies on Flores Island (Tucci et al., 2018), Greenland Inuit (Fumagalli et al., 2015) and Native Americans (Amorim et al., 2017; Harris et al., 2019), is also thought to be associated with dietary habits in cold climates, although the exact selection pressure is unknown (Fumagalli et al., 2015). An example is the similarity of genotypes in the genes of the olfactory system, which may play a role in the formation or maintenance of social bonds between individuals within a population (Christakis, Fowler, 2014). For such genotypes, higher rates of positive selection have been found (Fu et al., 2012).

The analysis of gene ontologies (according to WebGestalt, 3511 genes turned out to be annotated according to the gene ontology database) showed nine statistically significant biological processes (taking into account the Benjamini–Hochberg correction (FDR = 0.05)) associated with the detection of a chemical stimulus involved in the sensory perception of smell (GO:0050911, GO:0007608, GO:0050907, GO:0050906, GO:0051606, GO:0009593, GO:0007600, GO:0007606, GO:0050877).

An analysis of the literature also revealed that a number of genes that fall into IBD blocks can play a significant role in the formation of oncology and influence the treatment. For example, the AAGAB gene is included in the IBD blocks in five populations. AAGAB consists of 10 exons encoding a 315 amino acid (aa) protein, the AAGAB (alpha and gamma adaptin binding) protein. AAGAB is widely expressed and interacts with the gamma-adaptin and alpha-adaptin adapter protein complexes, AP1 and AP2. It is involved in membrane transport and plays a role in endocytosis and protein sorting. Heterozygous AAGAB mutations cause pitted palmoplantar keratoderma type 1 (PPKP1), a skin disease characterized by punctate hyperkeratosis of the palms and soles (Kiritsi et al., 2013). AAGAB is also a promising biomarker for chemotherapy response and outcome during breast cancer treatment. However, the exact role of AAGAB in the development of breast cancer is currently unclear and potentially requires further study (Bownes et al., 2019).

Another gene, GSE1, which falls into IBD blocks in five populations, may function as an oncogene in breast, stomach, and prostate cancer, and may also be important in the treatment of patients with prostate cancer (Bamodu et al., 2021).

IQCH-AS1 encoding antisense RNA IQCH 1 (IQCH-AS1) correlates with survival and diagnosis of cancer patients, but its role in the development of thyroid cancer and doxorubicin chemosensitivity remains unclear (Fei et al., 2022).

The role of SMAD3 in the regulation of genes important for cell development, such as differentiation, growth and death, implies that changing its activity or suppressing its activity can lead to the formation or development of cancer.

Also, some of the genes presented in Table 1 may play a role in human adaptation to environmental factors. For example, the IQCH gene may play a regulatory role in spermatogenesis (Yin et al., 2005) and is also associated with adult growth in Mongols (Kimura et al., 2008).

The CHRNA4 gene encodes for the α4β2 subcomponent of nicotinic receptors in the human brain. Individuals with certain CHRNA4 genotypes have been shown to be better at tracking and identifying multiple objects in visual search tasks (Espeseth et al., 2010). Polymorphisms in the CHRNA4 gene also seem to contribute to personality development by affecting the degree of developmental sensitivity to both normal and adverse environmental conditions (Grazioplene et al., 2013).

The COL20A1 gene encoding type XX alpha 1 collagen is noted in a number of genes with non-synonymous changes with a high frequency in modern humans compared to archaic hominids, which probably contributed to the development of unique human traits and is an interesting object for study (Kuhlwilm, Boeckx, 2019).

## Conclusion

Thus, as a result of our study, new information was obtained on the structure and composition of the gene pools of the indigenous peoples of Siberia, their genetic relationships and genetic and demographic processes based on an analysis of the distribution of linkage blocks identical in origin. The results obtained demonstrate the clustering of Siberian populations, linking peoples by place of origin, demonstrating a common origin and a high degree of kinship. The populations inhabiting the territory of Siberia are characterized by a unique genetic and demographic history, which is reflected in the distribution of IBD blocks both within the population and between them. The analysis of IBD blocks significantly complements the study of the formation and interaction of ethnic groups, but does not provide unambiguous answers for populations developing under conditions of complex ethnogenesis. With few exceptions, the overall IBD in Siberia is better explained by the geographical proximity of the populations rather than by their linguistic affiliation.

Analysis of SNPs that fell into more IBD segments of the analyzed populations made it possible to compile a list of 5358 genes. According to the results of calculations, biological processes enriched in these genes are associated with the detection of a chemical stimulus involved in the sensory perception of odor. Enriched with the found genes, molecular pathways are associated with fatty acid metabolism and olfactory transduction. At the same time, an analysis of the literature data showed that some of the selected genes, which were found in a larger number of IBD blocks in several populations at once, can play a role in human adaptation to environmental factors and are promising targets for further study.

## Conflict of interest

The authors declare no conflict of interest.
